# Modeling and Analysis of Acoustic Emission Generated by Fatigue Cracking

**DOI:** 10.3390/s22031208

**Published:** 2022-02-05

**Authors:** Weilei Mu, Yuqing Gao, Yuxue Wang, Guijie Liu, Hao Hu

**Affiliations:** 1 Engineering College, Ocean University of China, Qingdao 266100, China; muweilei@ouc.edu.cn (W.M.); gaoyuqing@stu.ouc.edu.cn (Y.G.); wangyuxue@stu.ouc.edu.cn (Y.W.); liuguijie@ouc.edu.cn (G.L.); 2 Suzhou Academy, Xi’an Jiaotong University, Suzhou 215123, China; 3 Qingdao Research Institute & School of Marine Science and Technology, Northwestern Polytechnical University, Xi’an 710072, China

**Keywords:** acoustic emission (AE), fatigue crack growth, predictive modeling

## Abstract

The acoustic emission (AE) method is a popular and well-developed method for passive structural health monitoring of metallic and composite structures. The current study focuses on the analysis of one of its processes, sound source or signal propagation. This paper discusses the principle of plate wave signal sensing using piezoelectric transducers, and derives an analytical expression for the response of piezoelectric transducers under the action of stress waves, to obtain an overall mathematical model of the acoustic emission signal from generation to reception. The acoustic emission caused by fatigue crack extension is simulated by a finite element method, and the actual acoustic emission signal is simulated by a pencil lead break experiment. The results predicted by the mathematical model are compared with the experimental results and the simulation results, respectively, and show good agreement. In addition, the presence of obvious S0 mode Lamb waves is observed in the simulation results and experimental results, which further verifies the correctness of the analytical model prediction.

## 1. Introduction

Today, many kinds of materials are used for construction infrastructure, aviation and ocean sailing. Applying the technical conditions of continuous monitoring to the security of these infrastructures is a challenge [1]. AE technology has shown great advantages in monitoring large structures, and it allows effective health monitoring and life prediction of materials. It is important to detect damage in the early stages to prevent catastrophes from occurring [2]. Mu et al. used visible graph modelling [3] and acoustic emission beamforming [4] to achieve AE source localization. In terms of material life prediction, Roberts et al. linked the AE count rate with crack growth [5] in an effort to predict the material’s remaining life. Similar methods have been used to link observed AE data trends with fracture [6] and crack growth [7] in metallic materials.

However, the AE electrical signal output by the AE transducer is often far from the real AE source signal [8]. In contrast with other nondestructive testing and evaluation methods for acoustics, AE lacks precise information related to the source, which complicates efforts to relate AE activity to, e.g., fracture location and size [9]. Therefore, determining how to obtain more information about the AE source, based on the electrical signal of the sensor, has become a challenge for the relevant practitioners.

Previous research on AE mostly uses statistical methods, such as duration, rise time, count and frequency, which reflect the physical characteristics of AE. W. Kaewwaewno et al. used AE detection technology to analyze the characteristics of valve leakage at low pressure, and the effect of differences in valve size and pressure on the effective voltage value of AE characteristic parameters [10]. Guo-Yang Ye et al. used Gaussian process regression (GPR) to develop a multivariate mathematical model to characterize the relationship between the AE signal and the pressure and leakage rate [11]. On the other hand, many researchers have proved that time–frequency conversion technology, as a very useful tool for AE signal analysis, can provide more reliable and comprehensive characteristic information about AE phenomena [12]. E. Mland et al. used kernel partial least squares regression (KPLSR) to establish the relationship between spectral components and leakage rate for both gas valves and liquid valves [13].

Another research method, analytical modeling of the AE signal, can be performed to predict the AE signal through the mathematical model. These studies help to understand the AE signal signature and source mechanism from both theoretical and experimental perspectives. Research on wave propagation and other basic theories is one of the development trends of AE technology [14].

Based on the theory of elastic dynamics and the dislocation model, Ohtsu and Ono developed the generalized theory of AE and the representation of the AE source mechanism in half-space [15], and further continued their work by performing a simulation of tensile cracks and shear cracks in half-space [16]. Researchers are also analyzing and studying the AE of the plate structure. Weaver and Pao [17] studied the numerical results of the surface response of a plate at different locations from the source for different modes of the Rayleigh–Lamb spectrum. Gorman and Prosser [18] studied the normal-mode solution to the classical plate bending equation to understand its applicability to AE. Aiming at the AE phenomenon caused by crack propagation, Lysak [19] studied the AE of growing cracks from the point of view of fracture mechanics, and described the models of crack initiation and subcritical growth in quasi-brittle materials. Giurgiutiu et al. [20] used a piezoelectric wafer active sensor (PWAS) to study the AE signal characteristics of fatigue crack growth in metal sheets.

For metallic or composite materials, the in-service conditions and failure modes are generally complex and may not be accurately predicted [21]. Guo-Yang Ye et al. [22] combined the time–frequency domain characteristics of acoustic emission signals and the random forest method to develop a multi-variable classification model that relates the internal leakage acoustic emission signal and the leakage rate under varying pressure. Ajay and Carlos [23] made efforts to accurately characterize guided wave excitation. They derived equations for the output voltage response of surface-bonded piezo-sensors in guided fields based on 3D linear elasticity equations. The above study focuses on the modeling of acoustic emission sources. Maillet and Morscher [24] proposed a new waveform-based procedure for localization of the AE source based on the Akaike information criterion (AIC). The energy-based approach also offers a potential for damage monitoring that could be used to improve the description of AE sources. Sause et al. [25] modeled the acoustic emission signal of hybrid multi-layer plates, focusing on the acoustic emission source and the signal propagation process. This model can solve the anisotropy problem in the plate, and it is more widely used. Sengupta et al. [26] proposed a refined 2D plate theory more applicable to plate structures, which satisfies the transverse shear stress continuity at the layer interface. This method has better computational efficiency compared to the 3D plate theory. These studies are mainly devoted to the modeling of acoustic emission signal propagation processes. Victor [27] described a model of the Lamb waves’ tuning mechanism with transducers. In this model, the piezoelectric wafer also used a thin adhesive layer to achieve structural coupling. Subsequently, he innovatively developed a theoretical model for the analysis of Lamb wave time reversal, and used it to analyze the Lamb wave propagation process [28]. His research focuses on the corresponding modeling of sensors. Zelenyak et al. [29] changed the geometry of the wave guide by varying the radius and height to investigate the influence on the detected signals. Similarly, this paper explores the effects of changes in the source, plate thickness and receiving sensor parameters on the detected signal. Unlike previous studies, the paper establishes a mathematical model of the overall process of the acoustic emission signal from generation and propagation to reception, and the accepted signal can be predicted based on the parameters of the source, propagation and reception.

In this paper, the AE induced by fatigue crack growth in a plate is analytically modeled by using a piezoelectric transducer. Section 3 and Section 4 verify the correctness of the analytical modeling using a simulation and an experiment, respectively. In Section 5, the factors influencing the spectrum of the received AE signal are discussed. The final part presents the summary and conclusions, and makes suggestions for further work.

## 2. Modeling Method of AE

The excitation in the fatigue crack growth process can be represented by the M11 dipole moment, and the dipole moment is used to deduce the complete solution of the wave field caused by AE.

**Figure 1:**
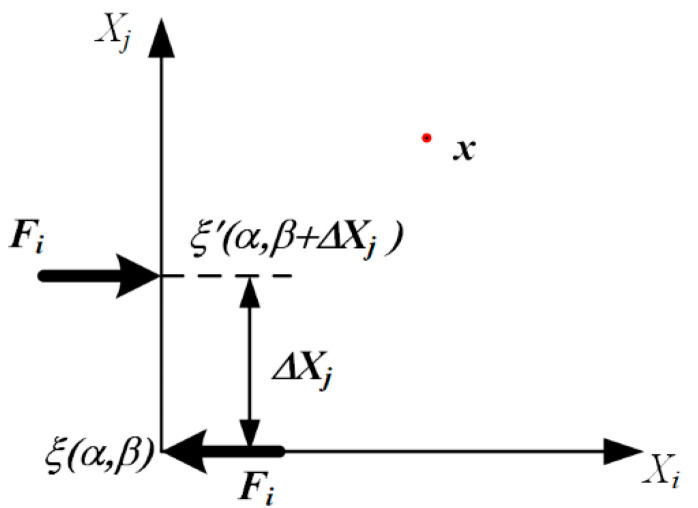
Schematic diagram of coupling force generating torque at small distances.

As shown in Figure 1, suppose the torque is generated due to the force vector $F_{i}$, applied at the position ξ(α,β), and the reverse force vector $F_{i}$ applied at the position $\xi^{\prime }\left(\alpha ,\beta +\Delta X_{j}\right)$, $\Delta X_{j}$ is a minute distance in the $X_{j}$ direction.

The displacement field at an arbitrary position *x*, due to the force vector $F_{i}$, is $u_{i}^{\xi }$ and $u_{i}^{\xi^{\prime }}$, respectively. The displacement field in the force couple $M_{ij}$ can be written as follows:

(1) $$u_{i}^{M_{ij}}=M_{ij}\frac{\partial }{\partial X_{j}}G\left(x;\alpha ,\beta \right)$$

where *G* is Green’s function of point forces and the force couple $M_{ij}=F_{i}\Delta X_{j}$.

**Figure 2:**
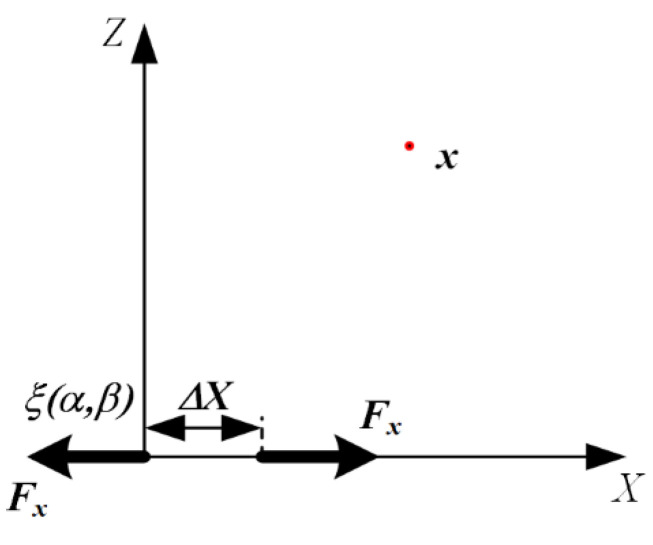
M11 dipole moment excitation.

In order to obtain the excitation field of the M11 dipole moment through the thickness of the plate, the dynamic reciprocity theorem was used to solve the elastic wave field generated by the force excitation through the thickness of the plate [30]. The M11 dipole moment excitation of the inertial plate thickness is shown in Figure 2, and the minute distance of the moment M11 is in the X direction.

The displacement of the wave field can be obtained as follows:

(2) $$\begin{array}{l} u_{M_{11}}=-ik_{n}M_{11}\sum_{n=0}^{\infty }\frac{U_{n}(h)}{2I_{nn}}U_{n}(z)\exp(-ik_{n}x)\\ w_{M_{11}}=k_{n}M_{11}\sum_{n=0}^{\infty }\frac{U_{n}(h)}{2I_{nn}}W_{n}(z)\exp(-ik_{n}x) \end{array}$$

where the harmonic factor exp(iωt) is omitted; parameter *n* represents the *nth* mode; $k_{n}$ represents the beam of the *nth* mode at a given frequency; h is half of the plate thickness; $\left[U_{n}\left(z\right),W_{n}\left(z\right)\right]$ is the mode shape, and its expression is $U_{n}^{S}=s_{1}\cos pz+s_{2}\cos qz$ and $W_{n}^{S}=s_{3}\sin pz+s_{4}\sin qz$; $T_{m}$ is the stress mode shape of the normal mode, and $I_{nn}^{}=\int_{-h}^{h}[T_{xx}^{n}U_{n}^{}(z)-T_{xz}^{n}W_{n}^{}(z)]dz$.

The strain can be obtained by the first derivative of Equation (2), as follows:

(3) $$\begin{array}{ll} \varepsilon_{{}_{M_{11}}}^{x} & =\frac{\partial }{\partial x}u_{M_{11}}=-k_{n}^{2}M_{11}\sum_{n=0}^{\infty }\frac{U_{n}(h)}{2I_{nn}}U_{n}(z)\exp(-ik_{n}x)\\ \varepsilon_{{}_{M_{11}}}^{z} & =\frac{\partial }{\partial z}w_{M_{11}}=k_{n}^{}M_{11}\sum_{n=0}^{\infty }\frac{U_{n}(h)}{2I_{nn}}W_{n}{}^{\prime }(z)\exp(-ik_{n}x)\\ & =k_{n}M_{11}\sum_{n=0}^{\infty }\frac{U_{n}(h)}{2I_{nn}}(s_{3}p\cos pz+s_{4}q\cos qz)\exp(-ik_{n}x) \end{array}$$

The PZT sensor is located on the plate. The PZT sensor detects the surface strain caused by the AE waveform and converts it to an equivalent voltage. Due to the physical characteristics of the PZT sensor, it does not significantly change the strain field of the incident wave. The voltage at both ends of the sensor electrode can be written as follows:

(4) $$V_{s}=-\int_{b/2}^{b/2+h_{s}}E_{z}\;dz=h_{s}\frac{e_{31}^{p}\varepsilon_{y}-D_{z}}{\in_{33}^{p}}$$

where b is the thickness of the plate and $h_{s}$ is the thickness of the sensor. $D_{z}$ is defined as the amount of charge per unit area.

The electrical boundary condition of the piezoelectric sensor is open circuit and the total charge on the electrode area is zero [31]. In the frequency domain, Equation (4) can be written as follows:

(5) $${\bar{V}}_{s}=C_{vs}\frac{1}{A}\int_{A}\bar{\varepsilon }dA$$

where A in Equation (5) represents the area where the PZT can generate charge. The electromechanical conversion coefficient can be defined as follows:

(6) $$C_{vs}=\frac{h_{s}e_{31}^{p}}{\in_{33}^{p}}=h_{s}\frac{e_{31}-e_{33}c_{13}/c_{33}}{\in_{33}+e_{33}^{2}/c_{33}}$$

**Figure 3:**
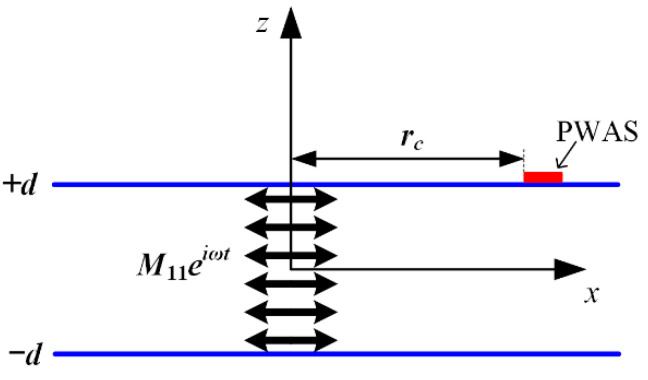
M11 excitation through plate thickness in thin plate.

The M11 dipole moment excitation through the plate thickness, as shown in Figure 3, is used to represent the dipole moment excitation generated by the type I crack. Therefore, the complete solution of the response of the M11 dipole moment excitation at the AE sensor is derived for the prediction modeling of the type I crack.

The distance between the PZT sensor and the AE source is $r_{c}$; $a_{s}$ is the radius of the PZT sensor. The traditional AE sensor mainly inducts the vibration of the structure to be measured by off-plane displacement. Therefore, the following equation can be obtained by substituting the strain of the excitation wave field of the dipole into the induction formula of the piezoelectric sensor:

(7) $$\begin{array}{ll} {\bar{V}}_{s}(x,\omega ) & =C_{vs}\frac{1}{A}\int_{A}k_{n}^{}M_{11}\sum_{n=0}^{\infty }\frac{U_{n}(h)}{2I_{nn}}W_{n}{}^{\prime }(z)\exp(-ik_{n}x)dA\\ & \overset{}{\underset{}{}}\underset{}{}{}_{}^{}\overset{}{\underset{}{}}=k_{n}^{}C_{vs}M_{11}\sum_{n=0}^{\infty }\frac{U_{n}(h)}{2I_{nn}}\frac{\sin k_{m}a_{s}}{k_{m}a_{s}}W_{n}{}^{\prime }(z)\exp(-ik_{m}x_{0})\\ & \overset{}{\underset{}{}}\underset{}{}{}_{}^{}\overset{}{\underset{}{}}=k_{n}^{}C_{vs}M_{11}\sum_{n=0}^{\infty }\frac{U_{n}(h)}{2I_{nn}}\frac{\sin k_{m}a_{s}}{k_{m}a_{s}}(s_{3}p\cos pz+s_{4}q\cos qz)\exp(-ik_{m}x_{0}) \end{array}$$

Translated to the time domain by the inverse Fourier transform, the following is obtained:

(8) $$\begin{array}{ll} V_{s}(x,t) & =\frac{1}{2\pi }\sum_{n=0}^{\infty }k_{n}^{}C_{vs}M_{11}\sum_{n=0}^{\infty }\frac{U_{n}(h)}{2I_{nn}}\frac{\sin k_{m}a_{s}}{k_{m}a_{s}}W_{n}{}^{\prime }(z)\exp(i\omega t)d\omega \\ & \overset{}{\underset{}{}}\underset{}{}{}_{}^{}\overset{}{\underset{}{}}=\frac{1}{2\pi }\sum_{n=0}^{\infty }k_{n}^{}C_{vs}M_{11}\sum_{n=0}^{\infty }\frac{U_{n}(h)}{2I_{nn}}\frac{\sin k_{m}a_{s}}{k_{m}a_{s}}(s_{3}p\cos pz+s_{4}q\cos qz)\exp(i\omega t)d\omega \end{array}$$

## 3. AE Simulation of Type I Fatigue Crack

### 3.1. Finite Element Simulation Settings

For validating the effectiveness of the analytical modeling, ANSYS software was used to conduct finite numerical analysis of the response precision of the piezoelectric sensor in the finite element model. The plate is set as 600 mm × 1.6 mm aluminum material, and is modeled by the two-dimensional unit Plan-182. The piezoelectric sensor is made of APC-850 material, and the two-dimensional multi-physical field unit Plan-13 is used for modeling. The size of the piezoelectric sensor is set as 20 mm × 0.42 mm. The characteristics of aluminum and piezoelectric materials are detailed in Table 1.

For the time-domain excitation of the dipole moment source, the wide-band cosine response function is used, and its mathematical representation is as follows [32]:

(9) $$E\left(t\right)=\left\{ \begin{array}{l} 0\begin{array}{cc} & t<0 \end{array}\\ 0.5\left(1-\cos\left(\pi t/\tau \right)\right)\begin{array}{cc} & 0\leq t\leq \tau \end{array}\\ 1\begin{array}{cc} & t>\tau \end{array} \end{array}\right.$$

where τ=1.5 μs is the rise time of the signal. The distance between dipoles is 0.2 mm.

**Figure 4:**
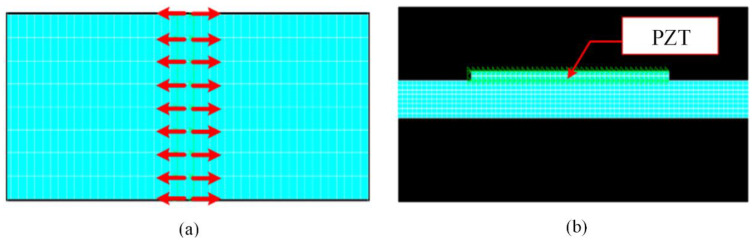
The simulation model: (**a**) dipole moment diagram; (**b**) overall model.

The excitation of the M11 dipole moment is located in the central position of the plate, and piezoelectric sensors are set on the upper and lower surfaces of the thin plate, 100 mm away from the AE source. The size of the grids is set as 0.1 mm × 0.5 mm. The simulation model is shown in Figure 4.

### 3.2. Verification and Analysis of Models

**Figure 5:**
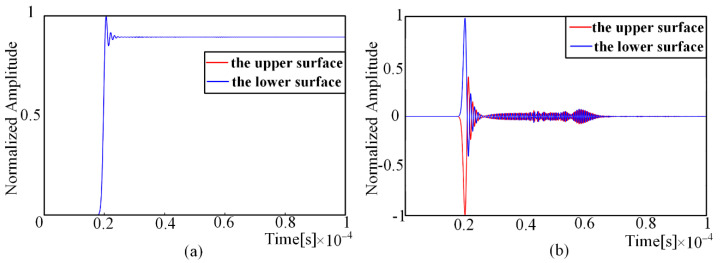
Displacement waveform of finite element simulation: (**a**) in-plane displacement; (**b**) off-plane displacement.

The in-plane and off-plane displacements of the nodes on the upper and lower surfaces of the thin plate were extracted at a distance of 100 mm from the excitation point, and the displacement waveform is shown in Figure 5. It can be observed that the in-plane displacements of the upper and lower surface nodes are in the same direction, while the off-plane displacements are in the opposite direction, which is in line with the particle motion characteristics of S0 mode Lamb waves.

**Figure 6:**
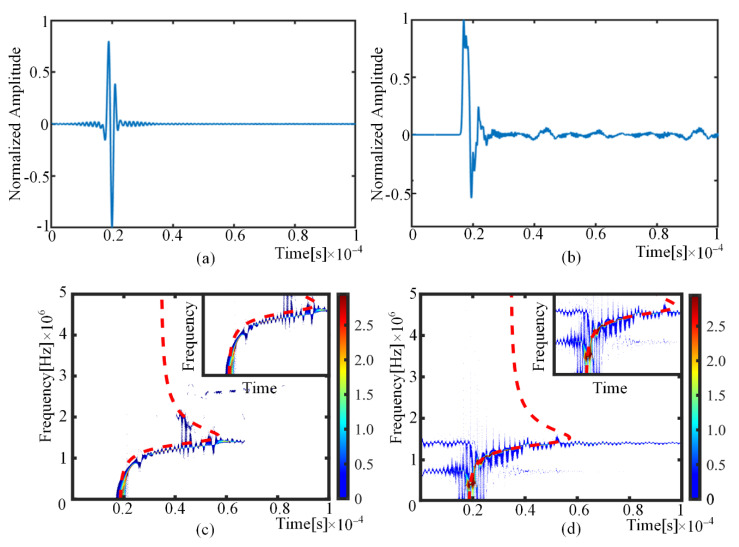
Sensor waveforms of finite element simulation and analytical prediction: (**a**) the analytical modeling result (time domain); (**b**) the finite element simulation results (time domain); (**c**) the analytical modeling result (short-time Fourier transform); (**d**) the finite element simulation results (short-time Fourier transform).

The results of the signal comparison between the finite element simulation and the prediction model are shown in Figure 6. As can be observed from the analysis results, Figure 6a,b show that the finite element simulation and analytical prediction results have a superb matching effect in the time domain. As the finite element model does not take into account issues such as wave absorption setting and high-frequency filtering, there are slight differences in the waveforms, which have little impact on the description of the AE phenomenon. Figure 6c,d show the results of the time–frequency analysis after the short-time Fourier transform of the time-domain waveform. It can be observed that the time–frequency analysis results have a high overlap with the time–frequency curve of the S0 mode Lamb wave. Type I crack excitation through the plate thickness only produces the S0 mode in the sheet pattern, and there is no A0 mode. The comparison of finite element simulations and theoretical simulations effectively proved the correctness of the analytical predictions.

## 4. Experimental Verification

### 4.1. Experimental Setup

**Figure 7:**
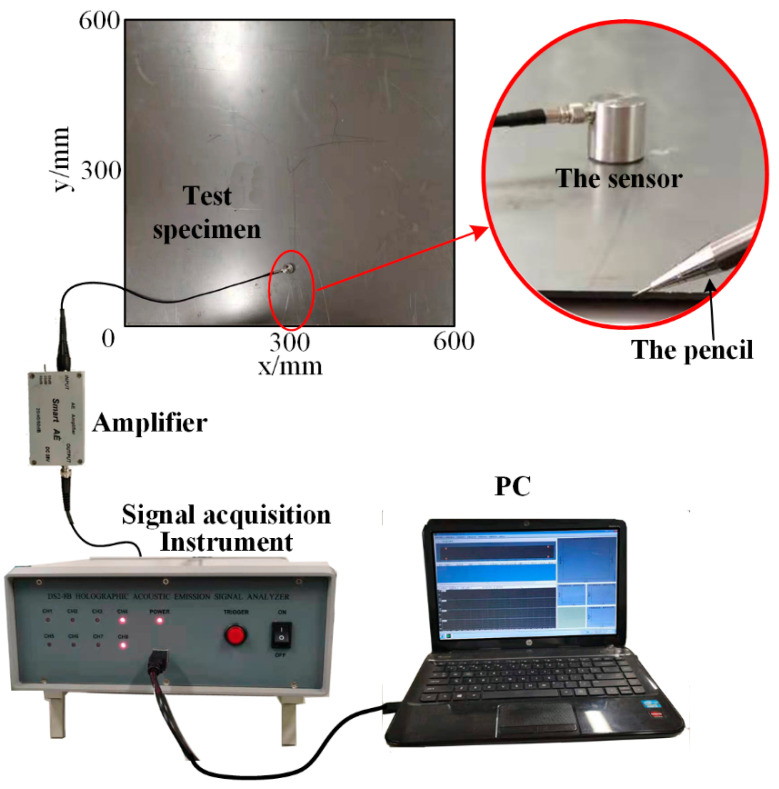
Experimental setup.

A square plate, with the dimensions 600 mm × 600 mm × 1.6 mm (length × width × thickness), was adopted as the experimental sample. The sensor was placed 10 mm away from the edge of the plate. The experimental setup consisted of a DS2−8B data acquisition instrument, a smart AE charge amplifier and a computer. The amplifier magnification was 10 times; the resonant frequency of the sensor was 150 kHz; a silicone coupling agent was used between the sensor and the plate. The experimental equipment and excitation details are shown in Figure 7.

It is impossible to accurately monitor the AE signal of simple type I crack growth in the laboratory environment. Based on the analysis of the crack and the load form, it can be concluded that the AE of type I crack growth can be simplified to the assumption of the load excitation in the inner surface of the thin plate. Therefore, a pencil lead break on the edge of the plate is used to simulate the AE phenomenon of the actual type I crack growth. It should be noted that the location of the broken lead should be, as far as possible, in the middle of the plate thickness to ensure the experimental effect.

### 4.2. Experimental Results

**Figure 8:**
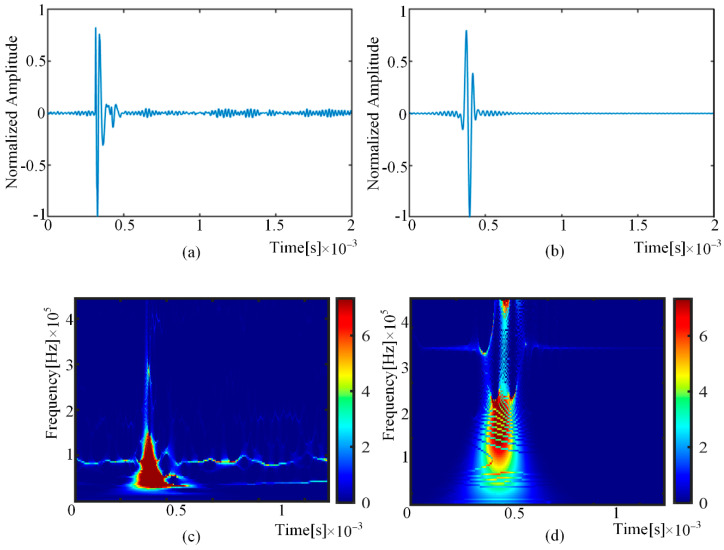
Comparison between the experimental result and the analytical modeling prediction result: (**a**) the waveform of experimental detection; (**b**) the waveform of analytical prediction; (**c**) experimental results (synchronous compression wavelet transform); (**d**) analytical prediction results (synchronous compression wavelet transform).

The experimental results are shown in Figure 8. It can be observed from Figure 8a,b that the experimental results and the analytical waveform have a good matching effect in the time domain. The time–frequency analysis results can be obtained through the synchronous compression wavelet transform of the time–domain waveform, as shown in Figure 8c,d. Because the frequency band of the pencil lead break is narrow, the analytical results and the experimental results are set at the same frequency band. The time–frequency diagram showed the waveform component as vertical linear distributed, which completely accords with the time–frequency performance of the S0 modal. Although the two spectra showed differences in details, this does not affect the verification of the correctness of the model.

**Figure 9:**
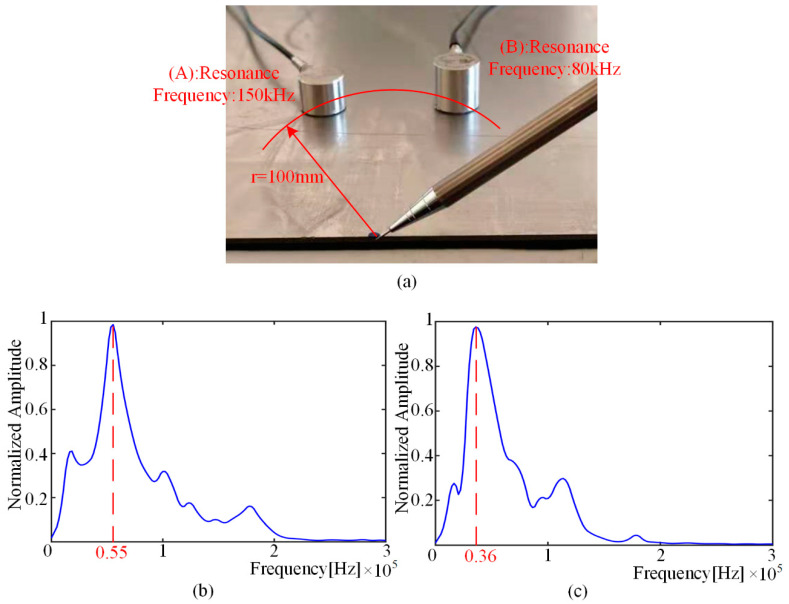
The experiment with sensors of different resonant frequencies: (**a**) experiment setup; (**b**) the frequency spectrum of the signal received by sensor A; (**c**) the frequency spectrum of the signal received by sensor B.

In the AE experiments, in addition to the obvious presence of S0 wave packets, the presence of slow-moving wave packets and a mass of interference noise can be observed. Since their frequency domain components differ significantly from the wave packets generated by AE, the frequency spectrum of the signal is affected. From Equation (8), it can be deduced that, for the same acoustic emission signal, the main frequency of the signal received by sensors with different resonant frequencies should also be different. As shown in Figure 9a, the sensors with resonant frequencies of 150 kHz and 80 kHz are arranged at the same distance from the excitation point. The frequency spectrum envelopes of the signals received by the two sensors are shown in Figure 9b,c. The experimental results are consistent with the predictions of analytical modeling.

## 5. Influencing Factors of AE Frequency Spectrum

The modeling of the AE monitoring process is divided into the following three components: the AE source response, the response of the plate structure, and the response of the sensor. These aspects will be discussed in this section.

### 5.1. Effect of AE Sources

**Figure 10:**
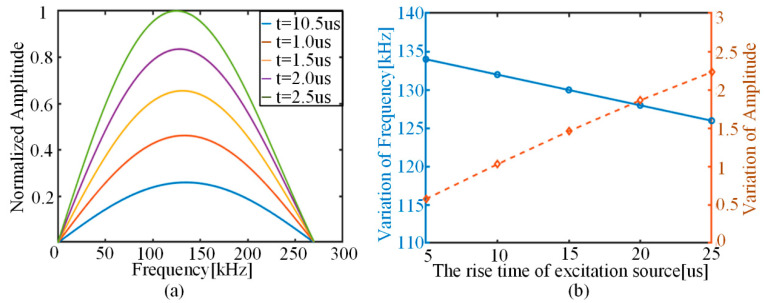
Variation in predicted AE results for different rise times: (**a**) change in model-predicted AE frequency spectrum; (**b**) trend of model-predicted main frequency and corresponding amplitude.

The AE source is modeled with reference to the dipole model, and the excitation function is a unified cosine clock function. Therefore, adjusting the rise time parameter τ of the function can effectively change the frequency of the excitation function, and, thus, obtain the best similarity between the experiment and the simulation. However, the actual AE signal cannot be measured directly, and the material properties, stress level and other factors may affect the AE signal frequency. By varying the rise time parameter *τ* of the cosine clock function in the prediction model, differences in the perceived AE signals under different rise time conditions are obtained, as shown in Figure 10. It can be observed that, with the increase in rise time τ, the main frequency of the AE sensing signal shows a decreasing trend, but the corresponding amplitude of the main frequency rises significantly. Although the main frequency and the corresponding amplitude basically show a linear change, the rise time of the function has a more significant effect on the main frequency amplitude, compared with the small change in the main frequency.

### 5.2. Effect of Structural Response

**Figure 11:**
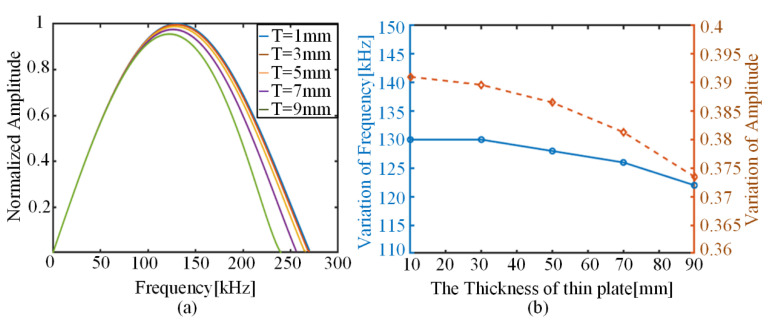
Variation in predicted AE results for different plate thicknesses: (**a**) variation in model-predicted AE spectrum; (**b**) trend of model-predicted main frequencies and corresponding amplitudes.

The role of the structural response in the AE prediction model is mainly reflected in the structural factor $U_{n}(h)W_{n}{}^{\prime }(z)/I_{nn}$. Analysis of the detailed expressions for the structural factor shows that the wave number k of the Lamb wave in the structure varies with the frequency component, in addition to the parameters related to the material properties of the structure. For an isotropic metal sheet with known material parameters, the frequency thickness product is directly related to the phase velocity of the Lamb wave. Therefore, the effect of wave number k on the frequency spectrum of the signal can be reflected by varying the thickness of the thin plate. The main peaks of the signal spectrum perceived by the piezoelectric sensor under different plate thickness conditions are shown in Figure 11. As the thickness of the thin plate increases, both the main frequency and the corresponding amplitude of the sensed signal show a decreasing trend, and the changes are relatively weak. Because the AE signal is mostly band-pass filtered at 30–700 kHz, and the S0 mode of the Lamb wave has a small phase velocity change in this frequency range, the corresponding wave number change is not obvious, and the change range is relatively small when reflected in the final spectrum.

### 5.3. Effect of Sensor Parameters

**Figure 12:**
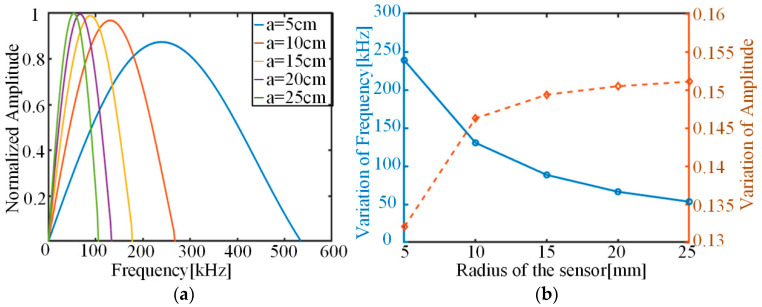
Variation in predicted AE results for different sensor radiuses: (**a**) variation in model-predicted AE spectrum; (**b**) trend of model-predicted main frequencies and corresponding amplitudes.

The electromechanical conversion coefficient $C_{vs}$ is related to the material properties of the piezoelectric material, and affects the amplitude of the signal. The parameter $\sin(k_{n}a_{s})/a_{s}$ of the sensor plays the role of signal frequency modulation. Considering that the wave number k does not vary much in this frequency range, the effect of the radius of the piezoelectric sensor is discussed. The main peaks of the signal spectrum received by piezoelectric sensors with different radiuses are shown in Figure 12. As the radius of the piezoelectric sensor becomes larger, the main frequency of the sensed signal decreases rapidly and the corresponding amplitude shows an inverse trend. It should also be noted that, unlike the effect of rise time, the sensor radius mostly affects the final signal main frequency, and the corresponding amplitude change is much smaller. This is related to the fact that the parameter $\sin(k_{n}a_{s})/a_{s}$ mainly plays the role of signal modulation.

## 6. Conclusions

This paper presents an analytical modeling method for AE caused by fatigue crack growth in a thin plate using a piezoelectric sensor. Assuming the existence of type I fatigue cracks in an isotropic thin metal plate, the dynamic analytical expression of Lamb wave propagation in the plate is derived by using the reciprocity theorem. On this basis, the concept of the dipole moment is used to model the type I fatigue crack growth AE source, and the constitutive equation of piezoelectric materials is combined. Finally, a complete analytical prediction model of the piezoelectric sensor AE signal caused by normal fatigue crack growth is obtained. By comparing the finite element simulations, experimental validation and analytical modeling, it is found that they are almost consistent in the time and frequency domains, and the presence of the Lamb wave S0 mode is clearly observed. These validate the accuracy of the analytical modeling predictions. The model is useful as a guide for the selection of detection equipment. The developed model allows the AE signal to be predicted in advance and prepared for the upcoming monitoring. For the same AE signal, different resonant frequencies of the sensor produce different main frequencies of the electrical signal. Therefore, when the ambient noise frequency is high, a sensor with a lower resonant frequency can be selected to distinguish the received AE signal from the noise.

This paper considers an ideal two-dimensional case and does not consider the effect of the third dimensional crack length and the difference in the three-dimensional propagation of guided waves in thin plates. Additionally, it only considers a type I open crack in the modeling process; the modeling of a slip-open crack and tear-open crack will be addressed in the future, as an extension of the current research. In the current study, the models of structural response and sensor response are relatively clear, while the crack expansion AE source is mostly simulated by the cosine clock function. In fact, there are differences in the signal spectrum at different stages of cracking. Therefore, it is important to investigate the relationship between the actual AE source and the stress level and material properties, and to construct a reasonable AE source simulation function. The above results obtained by AE analytical modeling still have certain simplifying conditions and sub-optimal considerations. For example, the signal modulation effect of the parameters is affected not only by the radius of the piezoelectric transducer, but also by the wave number *k*, which cannot be ignored. However, our conclusions are still important for the selection of piezoelectric transducers and the improvement in AE monitoring sensitivity.

## Figures and Tables

**Table 1:** Material parameters.

Material	Density (kg/m^3^)	Elastic Modulus (GPa)	Poisson’s Ratio
Aluminum plate	2700	69	0.33
APC-850	7700	84.3	0.31

## Data Availability

The supplementary data and simulation programs involved in this paper will be uploaded by the first author on the website of https://www.researchgate.net/profile/Weilei_Mu.
